# (*S*)-2-(Pyrrolidinium-2-ylmethyl­sulfan­yl)pyridinium dibromide

**DOI:** 10.1107/S1600536808008313

**Published:** 2008-05-24

**Authors:** Shuai Zhang, Yifeng Wang, Aibao Xia, Shuping Luo

**Affiliations:** a State Key Laboratory Breeding Base of Green Chemistry-Synthesis Technology, Zhejiang University of Technology, Hangzhou 310014, People’s Republic of China

## Abstract

In the title compound, C_10_H_16_N_2_S^2+^·2Br^−^, the pyrrolidine ring displays an envelope conformation, with the flap C atom lying 0.484 (5) Å out of the plane of the rest of the pyrrolidine ring. The thio­ether group connects the pyridine ring and the 2-methyl­pyrrolidine group. Both pyrrolidine NH bonds form hydrogen bonds to the bromide anions. These hydrogen bonds link the cations and anions in a helical chain along the *c* axis.

## Related literature

For related literature, see: Ishii *et al.* (2004 ▶); Xu *et al.* (2007 ▶); Larson (1970 ▶).
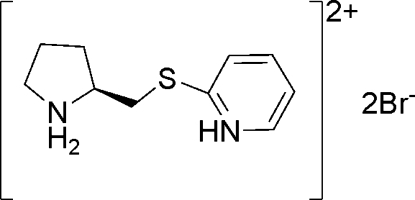


## Experimental

### 

#### Crystal data

C_10_H_16_N_2_S^2+^·2Br^−^
*M* *_r_* = 356.12
Trigonal, 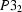

*a* = 8.9892 (9) Å
*c* = 15.4567 (14) Å
*V* = 1081.66 (18) Å^3^
*Z* = 3
Mo *K*α radiation
μ = 5.76 mm^−1^
*T* = 296 (1) K
0.35 × 0.30 × 0.23 mm

#### Data collection

Rigaku R-AXIS RAPID diffractometer
Absorption correction: multi-scan (*ABSCOR*; Higashi,1995 ▶) *T*_min_ = 0.162, *T*_max_ = 0.266
10585 measured reflections
3169 independent reflections
1902 reflections with *F* ^2^ > 2.0σ(*F* ^2^)
*R*_int_ = 0.061

#### Refinement

*R*[*F* ^2^ > 2σ(*F* ^2^)] = 0.036
*wR*(*F* ^2^) = 0.108
*S* = 1.01
3169 reflections
138 parameters
H-atom parameters constrained
Δρ_max_ = 0.67 e Å^−3^
Δρ_min_ = −0.53 e Å^−3^
Absolute structure: Flack (1983 ▶), 1037 Friedel pairs
Flack parameter: 0.017 (2)

### 

Data collection: *PROCESS-AUTO* (Rigaku, 1998 ▶); cell refinement: *PROCESS-AUTO*; data reduction: *CrystalStructure* (Rigaku/MSC, 2004 ▶); program(s) used to solve structure: *SIR97* (Altomare *et al.*, 1999 ▶); program(s) used to refine structure: *CRYSTALS* (Betteridge *et al.*, 2003 ▶); molecular graphics: *ORTEP-3 for Windows* (Farrugia, 1997 ▶); software used to prepare material for publication: *CrystalStructure*.

## Supplementary Material

Crystal structure: contains datablocks global, I. DOI: 10.1107/S1600536808008313/pk2087sup1.cif

Structure factors: contains datablocks I. DOI: 10.1107/S1600536808008313/pk2087Isup2.hkl

Additional supplementary materials:  crystallographic information; 3D view; checkCIF report

## Figures and Tables

**Table 1:** Hydrogen-bond geometry (Å, °)

*D*—H⋯*A*	*D*—H	H⋯*A*	*D*⋯*A*	*D*—H⋯*A*
N1—H2⋯Br1	0.86	2.45	3.278 (7)	163
N1—H3⋯Br1^i^	0.86	2.43	3.271 (5)	165

Symmetry code: (i) 
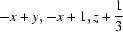
.

**Fig. 1.**
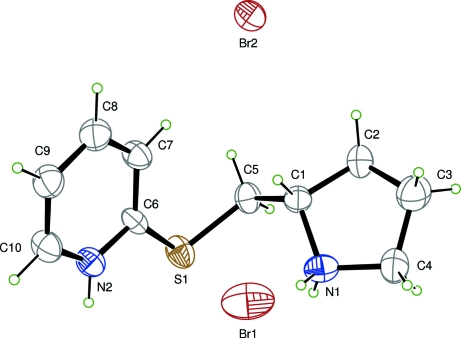
The asymmetric unit of the title compound, with the atomic labeling scheme. Displacement ellipsoids are drawn at the 40% probability level.

**Fig. 2.**
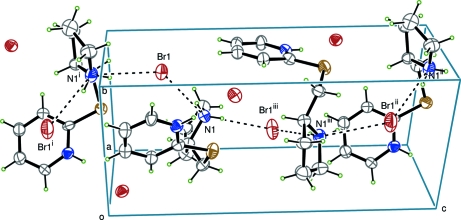
Hydrogen bonding in the title compound. Symmetry codes: (i) 1-y, 1+x-y, -1/3+z; (ii) 1-y, 1+x-y, 2/3+z; (iii) -x+y, 1-x, 1/3+z.

## supplementary crystallographic information

### Comment

In recent years, proline and its derivatives have been studied extensively because of their ability to catalyze a large number of reactions (Ishii *et al.*, 2004). The title compound is a hydrobromide of an ionic compound that was synthesized from *L*-proline. It was prepared as a kind of ionic organocatalyst for use in the asymmetric Michael addition of carbonyl compounds to nitroalkenes (Xu *et al.*, 2007). The compound consists of two ionic pairs, protonated ammoniums and Br^-^ anions. The chiral atom C1 has the expected S conformation, and the C1/C3/C4/N1 atoms of pyrrolidine are almost coplanar. The distance of atom C2 to the C1/C3/C4/N1 mean plane is 0.484 (5) Å, while the distance of atom C5 to the plane is 0.865 (9) Å. In addition, the dihedral angle of the C1/N1/C3/C4 mean plane and the pyridine ring is 67.82 (4) °. The thioether group connects the pyridine ring and the 2-methylpyrrolidine group, the torsion angle of C6—S1—C5—C1 is 97.13 (4) °.

### Experimental

The title compound was readily synthesized by treating 2-mercaptopyridine with (*S*)-(+)-2-bromomethylpyrrolidine hydrobromide in MeCN. The compound (*S*)-(+)-2-bromomethylpyrrolidine hydrobromide was obtained from commercially available *L*-proline by reduction with NaBH_4_ and subsequent bromination with PBr_3_. Suitable crystals were obtained by slow evaporation of methanol at room temperature.

### Refinement

All H atoms were placed in calculated positions with C—H=0.98 Å (*sp*), C—H=0.97 Å (sp2), C—H=0.93 Å (aromatic), N—H=0.86 Å and included in the final cycles of refinement as a riding model, with *U*_iso_(H)=1.2*U*_eq_ of the carrier atoms.

### Crystal data

**Table:**

C_10_H_16_N_2_S^2+^·2Br^–^	*Z* = 3
*M**_r_* = 356.12	*F*_000_ = 528.00
Trigonal, *P*3_2_	*D*_x_ = 1.640 Mg m^−^^3^
Hall symbol: P 32	Mo *K*α radiation λ = 0.71073 Å
*a* = 8.9892 (9) Å	Cell parameters from 5169 reflections
*b* = 8.9892 (9) Å	θ = 3.7–27.4º
*c* = 15.4567 (14) Å	µ = 5.76 mm^−^^1^
α = 90º	*T* = 296 (1) K
β = 90º	Chunk, colorless
γ = 120º	0.35 × 0.30 × 0.23 mm
*V* = 1081.66 (18) Å^3^	

### Data collection

**Table:**

Rigaku R-AXIS RAPID diffractometer	1902 reflections with *F*^2^ > 2.0σ(*F*^2^)
Detector resolution: 10.00 pixels mm^-1^	*R*_int_ = 0.061
ω scans	θ_max_ = 27.4º
Absorption correction: multi-scan(ABSCOR; Higashi,1995)	*h* = −11→11
*T*_min_ = 0.162, *T*_max_ = 0.266	*k* = −11→10
10585 measured reflections	*l* = −18→20
3169 independent reflections	

### Refinement

**Table:**

Refinement on *F*^2^	(Δ/σ)_max_ = 0.013
*R*[*F*^2^ > 2σ(*F*^2^)] = 0.036	Δρ_max_ = 0.67 e Å^−^^3^
*wR*(*F*^2^) = 0.108	Δρ_min_ = −0.53 e Å^−^^3^
*S* = 1.01	Extinction correction: Larson (1970) Crystallographic Computing eq. 22
3169 reflections	Extinction coefficient: 385 (18)
138 parameters	Absolute structure: Flack (1983), 1037 Friedel Pairs
H-atom parameters constrained	Flack parameter: 0.017 (2)
*w* = 1/[0.7600σ(*F*_o_^2^)]/(4*F*_o_^2^)	

### Special details

**Table:**

Refinement. Refinement using all reflections. The weighted *R*-factor (*wR*) and goodness of fit (*S*) are based on *F*^2^. *R*-factor (gt) are based on *F*. The threshold expression of *F*^2^ > 2.0 σ(*F*^2^) is used only for calculating *R*-factor (gt).

### Fractional atomic coordinates and isotropic or equivalent isotropic displacement parameters (Å^2^)

**Table:**

	*x*	*y*	*z*	*U*_iso_*/*U*_eq_	
Br1	0.51453 (17)	0.87503 (13)	0.16904 (9)	0.1032 (4)	
Br2	0.10004 (11)	0.14553 (10)	0.04190 (9)	0.0605 (2)	
S1	0.0678 (3)	0.4733 (3)	0.31433 (12)	0.0671 (7)	
N1	0.4515 (9)	0.5570 (8)	0.2963 (3)	0.067 (2)	
N2	0.0359 (7)	0.6865 (8)	0.2110 (3)	0.053 (2)	
C1	0.3184 (9)	0.4069 (9)	0.2462 (4)	0.053 (2)	
C2	0.3905 (11)	0.2858 (11)	0.2388 (5)	0.071 (3)	
C3	0.5771 (13)	0.4004 (16)	0.2379 (8)	0.086 (5)	
C4	0.6121 (14)	0.5513 (18)	0.2933 (7)	0.077 (5)	
C5	0.1434 (10)	0.3261 (10)	0.2879 (4)	0.059 (2)	
C6	0.0388 (9)	0.5412 (9)	0.2143 (4)	0.050 (2)	
C7	0.0160 (10)	0.4576 (10)	0.1352 (4)	0.059 (2)	
C8	−0.0040 (10)	0.5283 (11)	0.0612 (4)	0.066 (2)	
C9	−0.0037 (10)	0.6817 (12)	0.0635 (4)	0.067 (2)	
C10	0.0138 (10)	0.7576 (11)	0.1396 (4)	0.062 (2)	
H1	0.3105	0.4460	0.1880	0.064*	
H2	0.4673	0.6511	0.2735	0.080*	
H3	0.4187	0.5509	0.3491	0.080*	
H7	0.0144	0.3534	0.1330	0.071*	
H8	−0.0180	0.4724	0.0087	0.079*	
H9	−0.0154	0.7310	0.0130	0.080*	
H10	0.0108	0.8593	0.1432	0.074*	
H21	0.3560	0.2085	0.2879	0.085*	
H22	0.3522	0.2195	0.1857	0.085*	
H31	0.6169	0.4377	0.1793	0.103*	
H32	0.6343	0.3421	0.2614	0.103*	
H41	0.6450	0.5369	0.3511	0.092*	
H42	0.7033	0.6564	0.2682	0.092*	
H51	0.1477	0.2708	0.3409	0.071*	
H52	0.0614	0.2403	0.2484	0.071*	
H201	0.0494	0.7403	0.2590	0.064*	

### Atomic displacement parameters (Å^2^)

**Table:**

	*U*^11^	*U*^22^	*U*^33^	*U*^12^	*U*^13^	*U*^23^
Br1	0.1369 (10)	0.0925 (8)	0.0513 (4)	0.0357 (7)	0.0061 (5)	0.0034 (4)
Br2	0.0670 (6)	0.0510 (5)	0.0630 (4)	0.0292 (4)	0.0073 (4)	0.0054 (3)
S1	0.0848 (16)	0.0802 (16)	0.0554 (11)	0.0555 (14)	0.0125 (10)	0.0098 (10)
N1	0.072 (4)	0.063 (4)	0.053 (3)	0.026 (4)	−0.001 (3)	−0.006 (3)
N2	0.055 (4)	0.055 (4)	0.053 (3)	0.029 (3)	0.011 (2)	0.003 (2)
C1	0.052 (4)	0.046 (4)	0.058 (4)	0.022 (4)	−0.001 (3)	−0.001 (3)
C2	0.055 (5)	0.082 (6)	0.084 (5)	0.040 (5)	0.000 (4)	−0.005 (4)
C3	0.070 (8)	0.088 (10)	0.090 (14)	0.032 (7)	0.004 (7)	0.000 (10)
C4	0.061 (7)	0.083 (14)	0.093 (10)	0.041 (9)	−0.004 (6)	−0.003 (10)
C5	0.051 (5)	0.063 (5)	0.065 (5)	0.030 (4)	0.011 (3)	0.009 (3)
C6	0.056 (5)	0.041 (4)	0.060 (4)	0.030 (4)	0.008 (3)	−0.003 (3)
C7	0.075 (5)	0.056 (5)	0.055 (4)	0.040 (4)	0.002 (3)	0.002 (3)
C8	0.071 (6)	0.078 (6)	0.053 (4)	0.041 (5)	0.001 (3)	0.001 (4)
C9	0.077 (6)	0.082 (6)	0.053 (4)	0.049 (5)	−0.002 (3)	0.002 (4)
C10	0.088 (6)	0.061 (5)	0.050 (4)	0.048 (5)	0.002 (3)	0.009 (3)

### Geometric parameters (Å, °)

**Table:**

S1—C5	1.811 (11)	N1—H3	0.860
S1—C6	1.729 (7)	N2—H201	0.860
N1—C1	1.496 (8)	C1—H1	0.980
N1—C4	1.471 (18)	C2—H21	0.970
N2—C6	1.321 (12)	C2—H22	0.970
N2—C10	1.339 (10)	C3—H31	0.970
C1—C2	1.524 (16)	C3—H32	0.970
C1—C5	1.508 (10)	C4—H41	0.970
C2—C3	1.465 (12)	C4—H42	0.970
C3—C4	1.499 (9)	C5—H51	0.970
C6—C7	1.396 (9)	C5—H52	0.970
C7—C8	1.363 (12)	C7—H7	0.930
C8—C9	1.379 (16)	C8—H8	0.930
C9—C10	1.329 (10)	C9—H9	0.930
N1—H2	0.860	C10—H10	0.930
			
C5—S1—C6	103.5 (4)	C1—C2—H22	110.8
C1—N1—C4	108.0 (8)	C3—C2—H21	110.8
C6—N2—C10	125.8 (6)	C3—C2—H22	110.8
N1—C1—C2	104.4 (7)	H21—C2—H22	109.5
N1—C1—C5	112.6 (6)	C2—C3—H31	110.3
C2—C1—C5	113.7 (6)	C2—C3—H32	110.3
C1—C2—C3	104.2 (8)	C4—C3—H31	110.3
C2—C3—C4	106.2 (11)	C4—C3—H32	110.3
N1—C4—C3	106.5 (8)	H31—C3—H32	109.5
S1—C5—C1	115.3 (6)	N1—C4—H41	110.2
S1—C6—N2	117.7 (5)	N1—C4—H42	110.2
S1—C6—C7	126.9 (7)	C3—C4—H41	110.2
N2—C6—C7	115.4 (7)	C3—C4—H42	110.2
C6—C7—C8	120.2 (9)	H41—C4—H42	109.5
C7—C8—C9	120.7 (7)	S1—C5—H51	108.0
C8—C9—C10	118.4 (8)	S1—C5—H52	108.0
N2—C10—C9	119.5 (10)	C1—C5—H51	108.0
C1—N1—H2	109.8	C1—C5—H52	108.0
C1—N1—H3	109.8	H51—C5—H52	109.5
C4—N1—H2	109.8	C6—C7—H7	119.9
C4—N1—H3	109.8	C8—C7—H7	119.9
H2—N1—H3	109.5	C7—C8—H8	119.7
C6—N2—H201	117.1	C9—C8—H8	119.7
C10—N2—H201	117.1	C8—C9—H9	120.8
N1—C1—H1	108.7	C10—C9—H9	120.8
C2—C1—H1	108.7	N2—C10—H10	120.2
C5—C1—H1	108.7	C9—C10—H10	120.2
C1—C2—H21	110.8		
			
C5—S1—C6—N2	−159.4 (5)	N1—C1—C5—S1	51.7 (8)
C5—S1—C6—C7	21.3 (8)	C2—C1—C5—S1	170.2 (5)
C6—S1—C5—C1	66.8 (5)	C5—C1—C2—C3	−153.7 (7)
C1—N1—C4—C3	2.2 (10)	C1—C2—C3—C4	32.4 (11)
C4—N1—C1—C2	17.3 (7)	C2—C3—C4—N1	−22.0 (12)
C4—N1—C1—C5	141.1 (8)	S1—C6—C7—C8	−179.4 (6)
C6—N2—C10—C9	−1.5 (12)	N2—C6—C7—C8	1.2 (11)
C10—N2—C6—S1	−179.6 (5)	C6—C7—C8—C9	−0.6 (10)
C10—N2—C6—C7	−0.1 (9)	C7—C8—C9—C10	−1.0 (12)
N1—C1—C2—C3	−30.6 (8)	C8—C9—C10—N2	2.1 (12)

### Hydrogen-bond geometry (Å, °)

**Table:**

*D*—H···*A*	*D*—H	H···*A*	*D*···*A*	*D*—H···*A*
N1—H2···Br1	0.86	2.45	3.278 (7)	163
N1—H3···Br1^i^	0.86	2.43	3.271 (5)	165

Symmetry codes:  (i) −*x*+*y*, −*x*+1, *z*+1/3.
